# Patient-derived non-small cell lung cancer xenograft mirrors complex tumor heterogeneity

**DOI:** 10.20892/j.issn.2095-3941.2020.0012

**Published:** 2021-02-15

**Authors:** Xuanming Chen, Cheng Shen, Zhe Wei, Rui Zhang, Yongsheng Wang, Lili Jiang, Ke Chen, Shuang Qiu, Yuanli Zhang, Ting Zhang, Bin Chen, Yanjun Xu, Qiyi Feng, Jinxing Huang, Zhihui Zhong, Hongxia Li, Guowei Che, Kai Xiao

**Affiliations:** 1 National Chengdu Center for Safety Evaluation of Drugs and National Clinical Research Center for Geriatrics, West China Hospital, Sichuan University, Chengdu 610000, China; 2 Sichuan Kangcheng Biotechnology Co., Ltd. Chengdu 610000, China; 3 Department of Thoracic Surgery, West China Hospital, Sichuan University, Chengdu 610000, China; 4 GCP Center, West China Hospital, Sichuan University, Chengdu 610000, China; 5 Department of Pathology, West China Hospital, Sichuan University, Chengdu 610000, China; 6 Laboratory of Nonhuman Primate Disease Modeling Research, State Key Laboratory of Biotherapy, West China Hospital, Sichuan University, Chengdu 610000, China; 7 Center for Infectious Diseases, West China Hospital, Sichuan University, Chengdu 610000, China

**Keywords:** Patient-derived xenograft (PDX), non-small cell lung cancer (NSCLC), tumor heterogeneity

## Abstract

**Objective:**

Patient-derived xenograft (PDX) models have shown great promise in preclinical and translational applications, but their consistency with primary tumors in phenotypic, genetic, and pharmacodynamic heterogeneity has not been well-studied. This study aimed to establish a PDX repository for non-small cell lung cancer (NSCLC) and to further elucidate whether it could preserve the heterogeneity within and between tumors in patients.

**Methods:**

A total of 75 surgically resected NSCLC specimens were implanted into immunodeficient NOD/SCID mice. Based on the successful establishment of the NSCLC PDX model, we compared the expressions of vimentin, Ki67, EGFR, and PD-L1 proteins between cancer tissues and PDX models using hematoxylin and eosin staining and immunohistochemical staining. In addition, we detected whole gene expression profiling between primary tumors and PDX generations. We also performed whole exome sequencing (WES) analysis in 17 first generation xenografts to further assess whether PDXs retained the patient heterogeneities. Finally, paclitaxel, cisplatin, doxorubicin, atezolizumab, afatininb, and AZD4547 were used to evaluate the responses of PDX models to the standard-of-care agents.

**Results:**

A large collection of serially transplantable PDX models for NSCLC were successfully developed. The histology and pathological immunohistochemistry of PDX xenografts were consistent with the patients’ tumor samples. WES and RNA-seq further confirmed that PDX accurately replicated the molecular heterogeneities of primary tumors. Similar to clinical patients, PDX models responded differentially to the standard-of-care treatment, including chemo-, targeted- and immuno-therapeutics.

**Conclusions:**

Our established PDX models of NSCLC faithfully reproduced the molecular, histopathological, and therapeutic characteristics, as well as the corresponding tumor heterogeneities, which provides a clinically relevant platform for drug screening, biomarker discovery, and translational research.

## Introduction

Lung cancer (LC) is the leading cause of cancer deaths in men and women, with an estimated 2 million new cases and almost 1.7 million deaths occurring in 2016 worldwide^1^. According to the National Cancer Center (China), LC has been the most commonly diagnosed cancer and a major public health problem in the country since 2010, with more than 700,000 new cases and 600,000 deaths occurring in China every year^2,3^. Non-small cell lung cancer (NSCLC), which accounts for 85% of lung cancer cases, includes adenocarcinoma (ADC), squamous-cell carcinoma (SCC), and large-cell carcinoma (LCC)^4^. Despite significant improvements in the treatment of NSCLC in recent years, such as targeted therapy for specific genomic alterations, only moderate success has been achieved in the development of new antineoplastic drugs. Surgical resection remains the mainstay of treatment for the majority of patients with NSCLC, providing a poor 5-year survival rate. Therefore, there is an urgent need to improve treatments for NSCLC.

The emergence of precise therapies has led to significant progress in the war against cancer^5^. In 2017, Senft et al.^6^ reported precision medicine aimed to address inter- and intratumor heterogeneities, and to use multiple types of data to classify patients into groups that would most likely respond to a given treatment. However, precise medicine aims to address the heterogeneity between different individuals. Even the same pathological types of tumors have different responses to anti-cancer drugs. However, precision oncology is designed to overcome intratumor heterogeneity. Intratumor heterogeneity may facilitate tumor evolution and adaptation and hinder personalized approaches that depend on results from single tumor biopsy samples^7^. The complex tumor microenvironment is the leading cause of intratumor heterogeneity, in a setting where the tissue microenvironment provides the fitness selection for spatial and temporal changes in environmental pressures. Although tumor heterogeneity has an important impact on the efficacy and drug resistance of antineoplastic drugs, tumor heterogeneity and clonal evolution are very complex biological processes, and the progress of pathogenesis and treatment is still limited. Therefore, a better understanding of tumor heterogeneity is essential for precise treatments. With the rapid development of genotype-based individualized targeted therapies, preclinical trials using *in vitro* and *in vivo* models are essential in elucidating gene function and validating potential therapeutic targets^8^. In the last decade, a series of patient-derived xenograft (PDX) models have been developed and have rapidly gained favor over use of conventional cell lines as a preclinical drug screening platform, including for pancreatic cancer^9,10^, colorectal cancer^11–13^, hepatocellular carcinoma^14,15^, breast cancer^16,17^, epithelial ovarian cancer^18^, esophageal cancer^19,20^, and other cancers^21–23^. These PDX-based preclinical models accurately recapitulate the pathological and molecular characteristics of corresponding individual tumors, better reflecting patient heterogeneity and clinical diversity. Therefore, PDX, as a preclinical model relevant to humans, will facilitate the success of new drug development and drug repurposing, when compared with traditional cell line-derived xenografts^24^.

In the present study, we have established a panel of NSCLC PDX models (49.3%, 37/75) to validate their potential as a preclinical platform for precision medicine. The pathological, molecular, and pharmacological characteristics, as well as intra- and inter-tumor heterogeneities of representative PDX models were characterized in detail, and compared with the corresponding primary tumors. Our data demonstrated that the established PDX models of NSCLC were able to faithfully preserve the phenotypic and genetic features, drug responses, and complex tumor heterogeneities of the original tumors, which may provide an excellent preclinical platform to develop personalized and new therapeutic strategies using precision medicine.

## Materials and methods

### Patients and tissue specimens

Seventy-five fresh tumor specimens were obtained from patients with initially diagnosed NSCLC at initial surgery in the Department of Thoracic Surgery, West China Hospital, Sichuan University from 2015 to 2016. Immediately after surgery (within an average of 1 h after resection), surgical specimens were divided into 3 portions for the implantation into immunodeficient mice, DNA/RNA extraction, and pathological assessment. Written informed consent was obtained from each patient, and the study was approved by the ethics committee of West China Hospitals.

### Animals

All animal experiments were conducted in accordance with the Association for Assessment and Accreditation of Laboratory Animal Care guidelines, and protocols were approved by the Institutional Animal Care and Use Committee of the National Chengdu Center for Safety Evaluation of Drugs, West China Hospital, Sichuan University. Nonobese diabetic/severe combined immune deficiency (NOD/SCID) female mice (4–5-week-old) were purchased from Beijing Vital River Laboratory Animal Technology (Beijing, China). The mice were housed in individually ventilated cages at an appropriate temperature (21–25 °C) with a 12 h light/dark cycle and free access to food and water.

### PDX model establishment

Fresh clinical cancer specimens (3–5 mm^3^) were implanted subcutaneously into the flanks of 7–8-week-old mice, and collodiom was applied around the skin incision for wound healing. Each patient specimen was implanted into 5 NOD/SCID mice, which were then monitored for tumor growth for up to 150 days. When the tumor size was > 1 cm^3^, the PDX mice were anesthetized with 3% pelltobarbitalum natricum and the tumors were surgically removed, and tumor tissue not used for passaging, DNA/RNA extraction, or histopathological examination was cryopreserved for banking and later use.

### Histological staining

Histological staining of 4 μm formalin-fixed paraffin-embedded tissue sections was performed using an automated staining device (Benchmark XT; Ventana Medical Systems, Tucson, AZ, USA). On hematoxylin and eosin (H&E) staining, the pathological type and architecture between the original and xenograft tumors were reviewed by a pathologist to confirm the diagnosis. To compare the immunophenotypic characteristics, sample sections were incubated with various antibodies including anti-Ki-67 (1:400 dilution; Abcam, Cambridge, UK); anti-vimentin (1:200 dilution; Cell Signaling Technology, Danvers, MA, USA); anti-epidermal growth factor receptor (EGFR) (1:100 dilution; Cell Signaling Technology); and anti-programmed death-ligand 1 (PD-L1) (1:100 dilution; Abcam). The sections were subsequently incubated with secondary antibodies, and then visualized using a light microscope. Nuclei were counterstained with Harris hematoxylin.

### Reference-based transcriptome/RNA sequencing

Total RNA was extracted from tumor xenografts with TRIzol reagent (Invitrogen, Carlsbad, CA, USA) and genomic DNA was removed using DNase I (TaKaRa, Shiga, Japan). RNA quality was determined using a 2100 Bioanalyzer (Agilent Technologies, Santa Clara, CA, USA) and quantified using the ND-2000 (NanoDrop Technologies, Wilmington, DE, USA). Only high quality RNA samples were used to construct the sequencing library. A RNA-seq transcriptome library was prepared using a TruSeq™ RNA sample preparation kit (Illumina, San Diego, CA, USA) using 5 μg of total RNA. After quantitation using TBS380, a paired-end RNA-seq sequencing library was sequenced with the Illumina HiSeq xten (2 × 150 bp reading length). The raw paired end reads were trimmed and quality controlled by SeqPrep (https://github.com/jstjohn/SeqPrep) and Sickle (https://github.com/najoshi/sickle) with default parameters. The clean reads were then separately aligned to the reference genome with the orientation mode using TopHat (http://tophat.cbcb.umd.edu/, version 2.0.0) software^25^. The expression level of each transcript was calculated according to the fragments per kb of exon per million mapped reads (FRKM) method. RSEM (http://deweylab.biostat.wisc.edu/rsem/) was used to quantify gene abundances. R statistical package software EdgeR (Empirical analysis of Digital Gene Expression in R) (http://www.bioconductor.org/packages/2.2/bioc/html/edgeR.html) was used for differential expression analysis^26^.

### Whole exome sequencing (WES)

Genomic DNA of each sample was randomly broken into 150˜200 bp fragments for library construction using a TruSeq DNA sample preparation kit (Illumina). Libraries were pooled (500 ng each) for exome capture and amplification using the SureSelectXT Target Enrichment System for the Illumina Paired-End Sequencing Library, Illumina HiSeq, and MiSeq Multiplexed Sequencing Platforms, Protocol Version 1.3.1. Reads sequenced were then compared with refGene hg19 using BWA software, and sequenced reads generated by PCR-duplication were removed by Picard-tools. The mutation was annotated with Annovar software and hg19, and the annotation information of SNP and small InDel was obtained. SIFT and Ployphon-2 software was used to predict the potential mutations screened for amino acid replacement, to judge the impact of the mutations on protein structure and function. Copy number variations (CNV) profiles were generated from the BAM files using CopywriteR^27^. In short, sequence reads outside the captured genomic regions (off target reads) were used to generate DNA copy number profiles. A depth-of-coverage method was used for 100 kb bins, and the read count was normalized for GC content and mapping ability. Log_2_ ratios were calculated for all tumor samples. The normalized and corrected log_2_ ratios from CopywriteR were further analyzed by circular binary segmentation (CBS) and CGHcall (Bioconductor). CBS allows the detection of segments with nearly identical copy number states. CGHcall was used to classify data points as copy number gain, loss, or neutral.

### Preclinical efficacy of chemo-, targeted, and immunotherapeutics in PDX models

The LC-00536 and LC-00666 PDXs were used to test the response of NSCLC to chemotherapeutic and molecularly targeted drugs used in clinical practice as a standard adjuvant regimen. The LC-00536 patient was a 60-year-old female with stage IIIA and low differentiated squamous cell carcinoma (SCC) accompanied by PD-L1 gene amplification. The LC-00666 patient was a 71-year-old female with IB stage and moderately differentiated SCC, accompanied by mutations in the EGFR and FGFR genes. In brief, the chemotherapeutics paclitaxel [Mayne Pharma (Raleigh, NC, USA), 12.5 mg/kg), doxorubicin (DOX) [MCE (Bilovec, Czech Republic), 5 mg/kg], and cisplatin [Qilu Pharmaceutical (Shandong, China), 5 mg/kg] were administered intravenously into NSCLC PDX-bearing mice (*N* = 6 or 7) every 3 days with a total of 5, 6, and 6 doses, respectively. In addition, the FGFR targeting inhibitor, AZD4547 [Astra Zeneca (Cambridge, UK), 12.5 mg/kg] and the EGFR targeting inhibitor afatinib [Boehringer Ingelheim (Ingelheim am Rhein, Germany), 12.5 mg/kg] were administered intraperitoneally once a day for a total of 15 and 26 days, respectively. For the efficacy study of the PD-L1 antibody, 1 × 10^7^ human peripheral blood mononuclear cells collected from healthy volunteers were intravenously injected into PDX tumor-bearing NOD-SCID mice at 2 weeks before the drug administration. The PD-L1 antibody atezolizumab [GlaxoSmithKline (Brentford, UK), 10 mg/kg] was administered intravenously every 2 days for a total of 18 days. Mice in the control group were injected with the same volume of saline. The response to the therapy was evaluated by measuring the tumor volume of mice both in the treatment and the control groups.

### Statistical analysis

To determine clinical parameters that contributed to the establishment of PDXs, logistic regression analysis was conducted to evaluate the correlation between success rates and clinical pathological parameters. The Kaplan-Meier curve and log-rank test were used to evaluate the correlation between overall survival (OS), disease-free survival (DFS), and engraftment status. For the therapeutics study, data were analyzed by one-way analysis of variance for multiple comparisons, followed by Dunnett’s test for comparisons between 2 groups. A value of *P* < 0.05 was considered statistically significant.

## Results

### Establishment of a PDXs from NSCLC patient samples

We collected 75 tumor samples from 75 different NSCLC patients who had not been previously treated. The main clinical parameters of all patients are summarized in **Supplementary** **Table S1**. The ratio of males to females was 44:31 and the median age of patients at the time of surgery was 60 years. Of the 75 engraftments, 37 led to the successful establishment of PDX models, representing a tumor take rate of 49%. The clinical and pathological characteristics of these 37 patients are presented in **Table 1**. To determine the correlation between the successful rate of PDX implantation and clinicopathological parameters, the establishment rate of the PDX model was calculated and compared according to the characteristics of different patients (**Table 2**). A multivariate analysis showed that the histological subtype was the only parameter that significantly affected the success rate of PDX construction. PDX models of SCC were more likely to be successful in immunocompromised mice (17/23; 74%), compared with those of adenocarcinoma (ADC; 19/51; 37%). Other factors, including age, sex, smoking status, tumor size, pathological tumor node metastasis (TNM) stage, differentiation, and distant metastasis, did not correlate with the success rate.

**Table 1:** Clinical and pathological characteristics of 37 lung cancer patients and their tumors

Patient ID	Age (years)/gender	Smoking history (years)	Differentiation	TNM stage	Site	Histological cell type	Tumor size (cm^3^)	Metastasis
LC-00158	44/F	N	Moderately	IA	LUL	ADC	1.86	N
LC-00368	70/M	40	Poorly	IIB	RLL	SCC	18.85	N
LC-00536	60/F	N	Poorly	IIIA	RML	ADC	1.00	Y
LC-00666	71/F	N	Moderately	IB	LLL	SCC	31.41	N
LC-00592	62/M	40	Poorly	IIIB	RLL	ADC	126	Y
LC-00234	72/M	50	Poorly	IIB	RLL	SCC	81.73	N
LC-00576	66/M	45	Poorly	IA	RLL	ADC+SCC	2.25	N
LC-00088	52/M	20	Poorly	IB	RUL	SCC	25.13	Y
LC-00453	61/F	N	Poorly	IIIA	RLL	ADC	13.50	Y
LC-00374	76/M	30	Moderately	IB	RLL	SCC	14.14	N
LC-00178	73/M	30	Moderately	IB	RML	ADC	13.50	N
LC-00144	63/M	7	Moderately	IB	RLL	ADC	16.49	N
LC-00507	73/M	50	Poorly	IB	RUL	SCC		N
LC-00083	62/M	N	Poorly	IIIB	RML	SCC	108.00	Y
LC-00022	60/F	N	Moderately	IB	LUL	ADC	5.24	N
LC-00001	59/M	45	Moderately	IIA	LUL	SCC	108.00	N
LC-00827	70/M	50	Poorly	IIA	RLL	SCC	40.00	Y
LC-00877	58/M	35	Poorly	IB	LUL	SCC	32.00	N
LC-00781	61/M	40	Poorly	IIA	RLL	SCC	12.76	Y
LC-00343	59/M	40	Poorly	IIIA	LUL	SCC	12.76	Y
LC-00095	74/M	50	Poorly	IB	RLL	SCC	0.60	N
LC-00304	55/M	30	Poorly	IIIA	RLL	SCC	144	Y
LC-00702	62/M	45		IB	LLL	SCC	6.00	N
LC-00894	67/F	N		IA	RLL	ADC	4.19	N
LC-00010	55/F	N		IB	RUL	ADC	18.00	N
LC-00829	53/F	N		IA	LUL	ADC	4.19	N
LC-00813	66/M	45	Poorly	IIIA	RUL	ADC	65.45	Y
LC-00444	43/F	N		IB	LLL	SCC	13.74	Y
LC-00053	61/F	N			LUL	ADC	7.85	
LC-00700	62/M	30			RLL	SCC		
LC-00831	59/F	N	Moderately	IB	RLL	ADC	7.85	N
LC-00195	51/M	30	Poorly	IIIA	LLL	ADC	144.00	Y
LC-00167	52/M	30	Poorly	IIB	RUL	ADC	16.89	Y
LC-00576	62/F	N	Poorly	IA	LUL	ADC	15.08	N
LC-00615	55/F	N		IA	LUL	ADC	0.13	N
LC-00200	52/M	20	Well	IA	LUL	ADC	1	N
LC-00193	71/F	0	Poorly	IB	RUL	ADC	5.78	N

TNM, tumor node metastasis; ADC, adenocarcinoma; SCC, squamous cell carcinoma; LUL, left upper lobe; RLL, right lower lobe; RML, right middle lobe; LLL, left lower lobe; RUL, right upper lobe. The blank space indicates no information.

**Table 2:** Correlation between tumor take rate and patient clinical information

Parameters	Class	Tumor take rate (%)	*P*
Age (years)	< 60≥ 60	14/35 (40.0)23/40 (57.5)	0.632
Gender	MaleFemale	23/44 (52.3)14/31 (45.2)	0.548
Smoking status	EverNever	14/40 (35.0)21/35 (60.0)	0.074
Histologic subtype	ADCSCC	19/51 (37.2)17/23 (73.9)	0.015
Tumor size	< 11 cm^3^≥ 11 cm^3^	13/41 (31.7)22/30 (73.3)	0.077
Pathologic TNM stage	IIIIII	21/49 (36.0)5/10 (75.0)7/12 (36.0)	0.058
Distant metastasis	NY	22/51 (43.1)13/21 (61.9)	0.29
Differentiation	WellModeratelyPoorly	1/2 (50.0)8/22 (36.4)19/36 (52.8)	0.472

**Figure 1:**
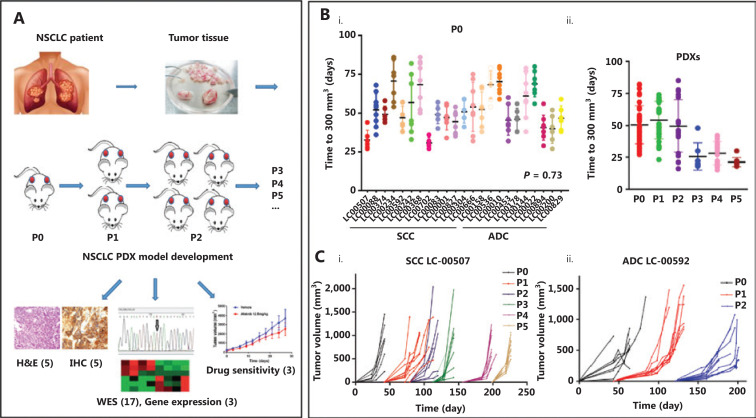
Patient-derived xenograft (PDX) tumor growth kinetics. (A) Illustration of non-small cell lung cancer PDX model development and characterization. (B) The scatter plot represents the time to tumor palpation (300 mm^3^) of the first generation (**i**) and passages 0–5 (ii) of 13 small cell carcinoma (SCC) PDXs and 10 adenocarcinoma (ADC) PDXs. (C) Tumor volume as a function of the days post-implantation of squamous cell carcinoma LC-00507 (i) and ADC LC-00592 (ii). Each color represents the specified generation and each curve represents a single tumor.

**Figure 1A** illustrates the process of the NSCLC PDX model construction, and its pathological, molecular and pharmacological characterizations. The tumor growth of implanted xenografts was monitored for up to 5 passages. It was noted that the time required for grafts from different patients to grow to 300 mm^3^ (early detectable tumor size) fluctuated between 27 and 86 days at the initial passage (P0, **Figure 1Bi**), and the histological subtype did not significantly contribute to engraftment (*P* = 0.73). However, the average growth time decreased to 25 days in passages 3–5 when compared with 50 days in passages 1–3 (**Figure 1Bii**). More importantly, the growth times between the fragments derived from the same original tumor tissue were also different. For example, xenografts originating from LC00242 PDX showed a maximum 50 day interval before growing to 300 mm^3^. In addition, an exponential growth trend was observed thereafter, illustrating the variation of tumor growth kinetics between PDXs (**Figure 1Ci and Cii**). The growth trends of xenografts from the same tumor tissue in specific passages were similar, but not entirely consistent.

**Figure 2:**
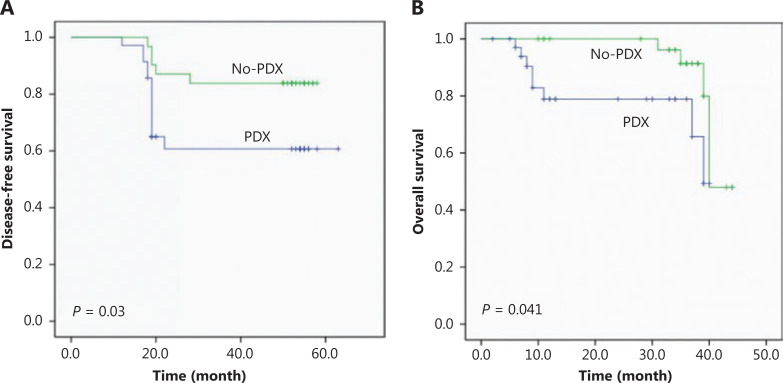
Disease-free survival (A) and overall survival (B) of non-small cell lung cancer patients according to the engraftment status of their patient-derived tumor xenografts using Kaplan-Meier plots and the log-rank test.

To determine the correlation between implant outcome and clinical prognosis, we analyzed the OS and DFS of the NSCLC patients recruited in this study. Patients with successful tumor engraftment had a significantly shorter DFS (*P* = 0.03) and OS (*P* = 0.041) than those without successful PDX construction (**Figure 2A and 2B**). Among the patients with successful PDX construction, those with an SCC had a trend of inferior DFS (*P* = 0.155) than those with an ADC, and patients with an SCC and no PDX establishment had a worse DFS (*P* = 0.046) than those with an ADC (**Supplementary Figure S2A**). However, the OS between SCC and ADC was not significantly different either in patients with or without successful PDX construction (**Supplementary Figure S2B**).

### Histopathological fidelity between patient tumors and corresponding PDXs

**Figure 3:**
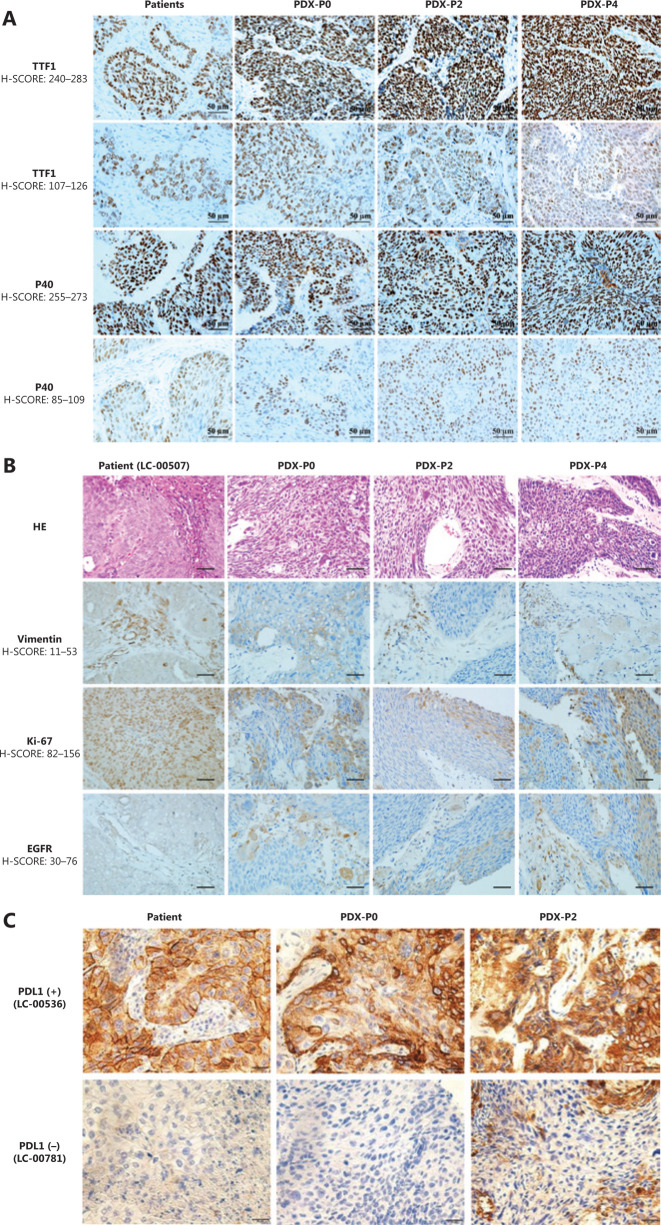
Histopathological characterization of patient primary tumors and corresponding xenografts. (A) Representative immunohistochemical staining for TTF1 and P40. (B) Representative hematoxylin and eosin staining and immunohistochemical staining for vimentin, Ki-67, and EGFR of LC-00507. (C) Immunohistochemical staining for PD-L1 of LC-00536 and LC-00781 (400×).

To evaluate whether the established PDXs retained histological characteristics and whether the expression pattern of biomarkers was consistent with the tumor of origin, histopathological and immunohistochemical examinations were performed by staining with H&E and clinically relevant biomarkers, including the primary markers for adenocarcinoma (TTF1), squamous (P40) (**Figure 3A**), vimentin, Ki67, and EGFR and PD-L1 (**Figure 3B**). The results showed that the morphology and immunophenotype of PDX tumors were similar to those of patients from which the primary PDX models were derived. Consistently, PDX derived from PD-L1 positive (LC-00536) or negative (LC-00781) patients retained the intertumor heterogeneities (**Figure 3C**). Notably, a fair degree of tumor stroma was retained throughout passaging but did appear to decrease when compared with that observed in the primary tumors. Overall, these observations confirmed that PDX collection recapitulated the main histological and immunophenotypic profiles of the corresponding NSCLCs.

### Molecular heterogeneity of patient tumors and corresponding PDXs

#### RNA sequencing

**Figure 4:**
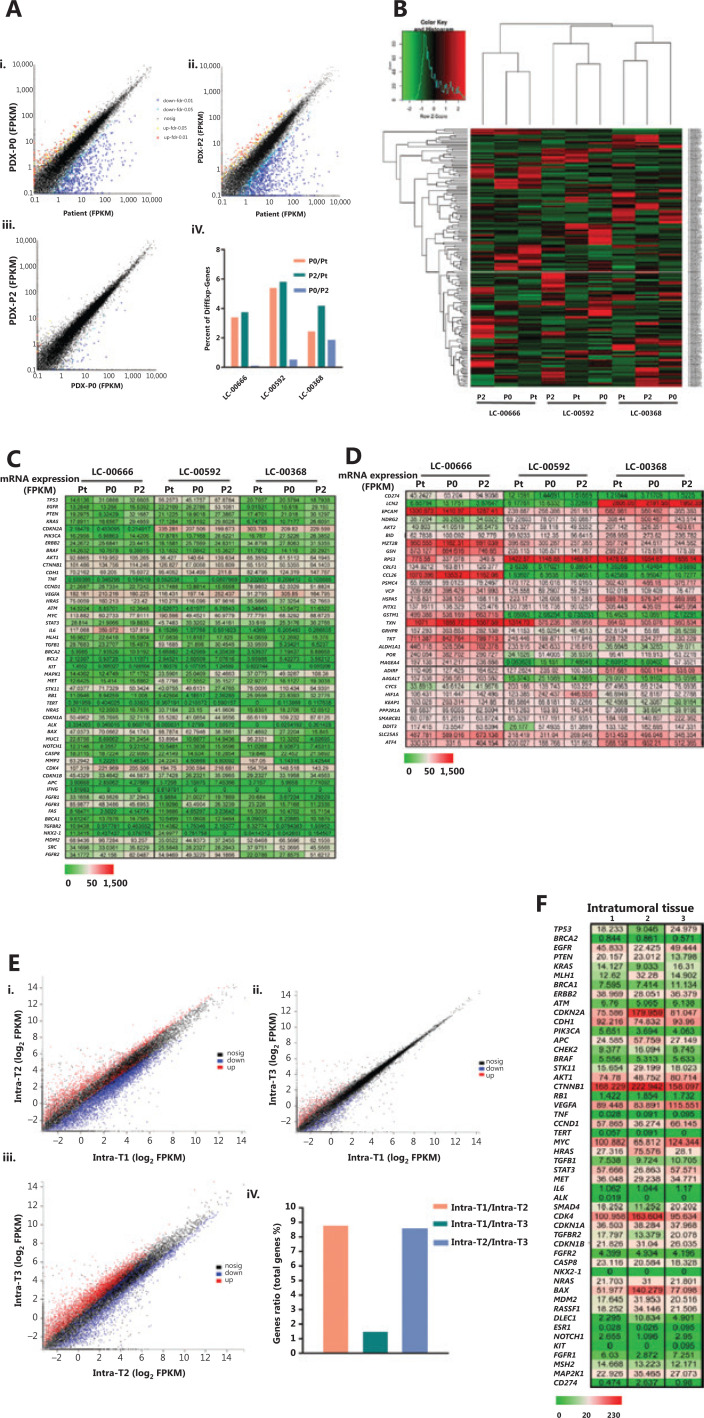
Gene expression analysis between primary patient tumors and patient-derived xenographs (PDXs). (A) Comparison of genome-wide gene expression profiles between matched patient primary non-small cell lung cancer (NSCLC) tumor (patient LC-00666), the corresponding passage 0 (P0) and passage 2 (P2) of xenografts (i, ii, iii). Percent of differentially expressed genes in 3 PDX matched groups (LC-00666, LC-00592, and LC-00368) (iv). (B) Heat map of cancer-related gene expressions in 3 PDX matched groups. The dendrogram shows unsupervised hierarchical clustering of samples according to gene expression patterns. (C) Gene level expression (FPKM) values for the top 50 oncogenes related to NSCLC, and (D) Another 32 differentially expressed oncogenes are presented by the heat map (green = lower than the median, red = greater than the median). (E) Comparison of genome-wide gene expression profiles and (F) NSCLC-related gene expression (FPKM) values between three pieces of intratumoral tissues from one PDX (P4 of LC-00536).

To establish the correlation between gene expression profiles of primary and xenograft tumors, we first performed a pilot study in three randomly selected PDX matched groups and analyzed the differentially expressed genes. Each group was comprised of the original patient tumors, and the corresponding passage 0 and passage 2 of PDX xenograft tumors (Pt, P0, and P2). The results showed that compared with the original tumors of LC-00666, LC-00592, and LC-00368, among 60,662 genes, the number of differentially expressed genes in P0 was 2,082, 3,299, and 1,509, and that in P2 was 2,298, 3,558, and 2,567, respectively, with an average fold change of less than 6%. In addition, the number of differentially expressed genes between P0 and P2 was 78,333, and 1,151, respectively, with an average fold change of less than 2% (**Figure 4A**). Consistently, unsupervised clustering of cancer-related gene expressions (488 genes) showed that all of the three PDXs clustered tightly together with the corresponding patient tumors (**Figure 4B**). These results demonstrated that PDX authentically maintained the gene expression characteristics of the patient tumors.

To identify the genetic stability of PDXs, we selected the top 50 oncogenes related to NSCLC from the GeneCards Database (https://www.genecards.org) and analyzed gene expressions between the original tumors and xenografts. Not surprisingly, for each PDX, gene expressions in P0 and P2 were consistent with the primary tumors. More importantly, the expression of some genes such as *CDKN2A*, *CCND1*, and *IL6* varied among different patients, and this difference was well-preserved in the corresponding PDX (**Figure 4C**). Another 32 differentially expressed oncogenes further demonstrated that genetic heterogeneity was precisely maintained in PDXs, including cell immunity (*CD274, MAGEA4*), invasion (*LCN2*), differentiation (*EPCAM, DNRG2, MZT2B*), apoptosis (*BID, RPS3*), protein degradation (*VCP, HSPA5,PSMC4*), and redox (*GRHPR*, *TKT*, *POR*) (**Figure 4D**). These results showed that PDX accurately replicated the intertumor molecular heterogeneities of clinical tumors.

To further investigate whether PDXs retained intratumor heterogeneities, we randomly isolated 3 pieces of intratumoral tissues from 1 PDX tumor (P4 of LC-00536) and detected gene expression profiles. The results showed a fair degree of differentially expressed genes in 3 different tumor tissues when compared with each other, with an average fold change of 1%–9% (**Figure 4E**). Consistently, no significant difference was observed in the expressions of most oncogenes, while a panel of oncogenes, such as* CDKN2A*,* MYC*, *CDK4*, and *BAX*, were differentially expressed in intratumoral tissues (**Figure 4F**). These results showed that PDX was an ideal animal model for intratumor heterogeneity studies.

#### WES analysis

To further investigate whether the tumors in PDX reflected patient heterogeneity and to gain insights into genomic molecular alterations of NSCLC, we performed whole genome sequencing on the P0 xenograft tumors from 17 PDX models.

**Figure 5:**
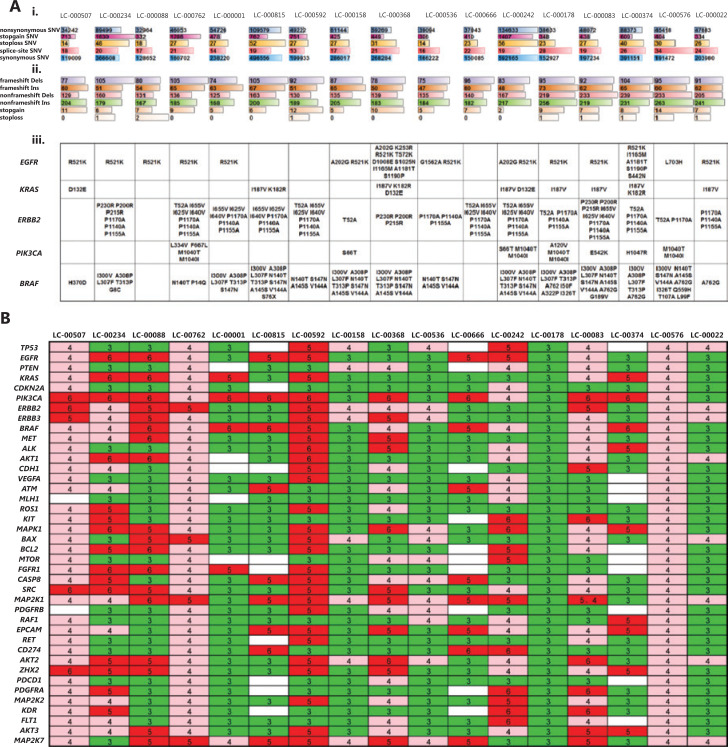
Whole genome sequencing of P0 xenografts from 17 patient-derived xenograph models. (A) Single nucleotide variants (SNV), insertions (Ins) and deletions (Dels) mutations in the exon genome. The type of alteration is indicated by different colors, and bar length represents the number of SNV and Ins/Dels (i, ii). The mutation status (amino acid changes) is shown for a panel of genes that are currently of interest in routine practice for non-small cell lung cancers (iii). (B) Gene copy numbers are presented in a heat map, and variants indicated as gains (i.e., > 2) are shown by shades of green ( = 3), pink ( = 4), and red (> 5).

We detected a median of 454,264 (range: 238,520–902,439) somatic SNV alterations in P0 of 17 PDX models. A minimum of 32,964 nonsynonymous somatic SNVs were identified in LC-000888, including 332 stopgain and 18 stoploss, compared with a maximum of 134,633 nonsynonymous somatic SNVs in LC-000242, including 1,407 stopgain and 56 stoploss. A total of 348 splice altering SNVs were also detected (range: 13–34) (**Figure 5Ai**). In addition, the median number of nucleotide insertions and deletions (INDELs) detected in P0 of 17 PDX models was 10,466 (range: 7,031–19,413). A minimum of 122 nucleotide INDELs resulting in frameshifts were detected in LC-000536, including 319 non-frameshift variants, 5 stopgain and non-stoploss, compared with a maximum of 172 nucleotides INDELs frameshifts detected in LC-000815, including 363 non-frameshift variants, and 6 stopgain and non-stoploss (**Figure 5Aii**).

Additionally, we analyzed gene mutations of target genes in lung cancer-targeted therapy and we found that many broad and focal gene mutations were shared. Among the 17 PDXs, we detected a total of 12 *EGFR* mutations in 14 models, especially 13 R521K and 3 A202G mutations.* KRAS* mutations were observed in 8 models, including 7 I187V, 3 K182V, and 3 D132E mutations. In addition, both *PIK3CA* M1040T and M1040I mutations were detected in 4 models. Three *ERBB2* mutations, including P1170A, P1140A, and P1155A, were detected in 12 PDX models (**Figure 5Aiii**).

Another result of our analysis was the association of a large number of CNA in NSCLCs with intertumor heterogeneities. In the 17 PDX models, different genes had different CNVs in each patient-derived PDX, and the CNV of the same gene also varied among all PDXs. Of the 40 genes highly associated with NSCLC, at least 4 copies of every gene were detected, among them *BRAF* and *MAP2K1* had a maximum of 74 CNVs in all 17 PDXs, compared with a minimum of 45 CNVs of *MLH1*. In addition, a maximum of 180 CNVs were identified in LC00592, compared with a minimum of 110 CNVs in LC-00666 and LC-00374 (**Figure 5B**). Together, these results showed that PDX preserved the heterogeneity of genetic mutations and copy number variations among different patients.

### Efficacy validation of standard-of-care therapies in NSCLC PDX models

**Figure 6:**
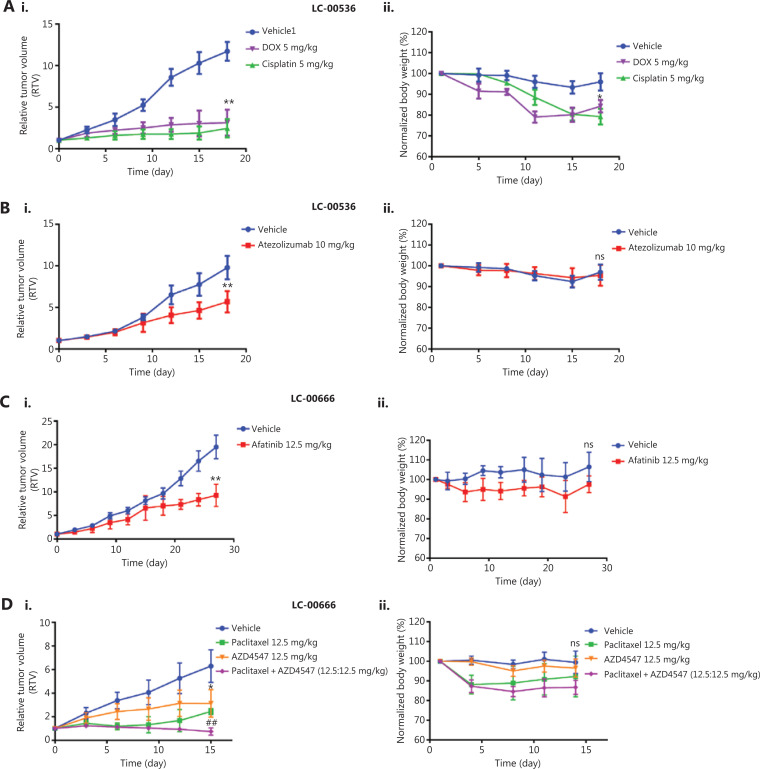
Efficacy validation of chemotherapeutics and molecularly targeted therapeutics. Changes in relative tumor volume and body weight after (A) A patient-derived xenograph (PDX) (LC-00536) was treated with doxorubicin, cisplatin, and (B) Atezolizumab for 18 days. (C) A PDX of LC-00666 was treated with afatinib for 27 days, and (D) PDX LC-00666 was treated with paclitaxel, AZD4547, and paclitaxel plus AZD4547 for 15 days when tumors reached 100˜200 mm^3^ post-implantation. *P < 0.05; **P < 0.01 *vs*. vehicle; ^##^*P* < 0.01 *vs.* paclitaxel or AZD4547; ns, no significant differences *vs*. vehicle.

We selected two PDX models derived from LC-00536 and LC-00666 to evaluate their responses to the clinical standard-of-care agents and investigational new drugs, respectively. We first validated the therapeutic efficacies of several clinically used chemotherapeutics (doxorubicin, paclitaxel, and cisplatin) in NSCLC PDX models. The results showed that all chemotherapeutic drugs significantly inhibited PDX tumor growth (**Figure 6Ai and Di**, *P* < 0.05). However, the mice in the doxorubicin and cisplatin treatment groups experienced significant weight loss (**Figure 6Aii and Dii**), which was consistent with the high toxicity of these chemotherapeutic drugs in clinical treatment. To elucidate the application of the molecularly characterized PDX models in precision medicine, we first selected the PD-L1 antibody, atezolizumab, and the EGFR inhibitor, afatinib, for targeted therapy of LC00-536 (PD-L1 overexpression) and LC-00666 (EGFR mutation), respectively. The results showed that both targeted drugs were effective in PDX models with corresponding genetic alterations (**Figure 6Bi and 6Ci**), without significant toxicities (**Figure 6Bii and Cii**). We also evaluated the therapeutic efficacy of an FGFR inhibitor, AZD4547 (currently in phase III clinical trials) in two NSCLC PDX models; our results showed that PDX LC-00666 (overexpression of FGFR) was sensitive to AZD4547 treatment (**Figure 6Di**), while PDX LC-00536 (low expression of FGRF) showed almost no response (**Supplementary Figure S1Ai**). In addition, combination with paclitaxel further improved the therapeutic efficacy of AZD4547 in the PDX LC-00666 model with no significant increase in toxicity (**Figure 6Di and 6Dii**), suggesting that the combination of AZD4547 with paclitaxel may have great potential in the treatment of NSCLC. These results indicated that these NSCLC PDX models responded to the stand-of-care agents similar to the clinical results, and showed differential efficacy in different subgroups of cancer patients representing tumor heterogeneities.

## Discussion

Establishing an appropriate preclinical model is crucial for translational cancer research. The PDX model has shown advantages as a preclinical model in drug screening, biomarker development, and co-clinical trials. In this study, we successfully established a total of 37 NSCLC PDX models. More than 55 additional clinical lung cancer samples, including small cell lung cancer, multiple primary lung cancer, and EGFR-TKIs resistant lung cancers, are being constructed as PDX models in our laboratory. Our PDX model bank of lung cancers is a critical tool for follow-up research, such as in studies of tumor microenvironments, drug resistance, and diagnostic biomarkers.

Among the large variety of tumors transplantable into immuno-deficient mice, LC is in the most need of establishing PDX models^28–33^. Different reasons can explain this. (a) LC is characterized by high morbidity and mortality. Furthermore, it poses a serious threat to human health and quality of life, so researchers have been working to develop a model for LC that mimics the clinical environment, which can be used to understand the impact of various anti-cancer treatments. (b) There are numerous types of lung cancer. It is difficult to simulate all types of lung cancer with traditional animal models, especially multiple primary lung cancer and metastatic lung cancer. However, PDX can overcome the limitations of traditional models, thus providing a stable and reliable preclinical model for studying the pathogenesis, progression, recurrence, and metastasis of various types of lung cancers. (c) There is a rapid development of anti-lung cancer drugs. The development of anti-tumor drugs has undergone many changes in the past 20 years. In particular, the emergence of targeted therapy is a milestone for the development of personalized cancer therapy^5,34^. More than 20 drugs have been approved by the Food and Drug Administration (FDA) as treatments for NSCLC, including 14 targeted and immunotherapeutic drugs, such as the anti-EGFR antibody, cetuximab, the EGFR and HER2 inhibitors, afatinib, the PD-L1 inhibitor, atezolizumab, and the vascular endothelial growth factor antibody, bevacizumab. However, due to mounting drug resistance, existing drugs are far from meeting the clinical needs of NSCLC patients. New targets need to be discovered, and new drugs need to be accurately screened and evaluated in the PDX platform in view of its consistency with patient response in predicting drug efficacy^28,30,35^. (d) Lung cancer is highly susceptible to drug resistance. Most NSCLCs will develop drug resistance within a year of treatment, including to chemotherapy and targeted therapy. For example, the third-generation EGFR-TKI, osimertinib (AZD9291) was approved by the FDA for the treatment of EGFR-TKIs-acquired T790M resistance NSCLC in 2015, followed by a report in 2016 about the fourth-generation targeted drug EAI045 that overcomes AZD9291 resistance^36^. The PDX can predict resistance mechanisms, provide preclinical solutions to overcome resistance, and promote the development of next-generation drugs. Our established NSCLC PDX models combined with clinical and genomic annotations could be utilized to validate new therapeutic regimens, elucidate the mechanism of disease progression or drug resistance, clarify the heterogeneity between primary and metastatic tumors, and ultimately trace the dynamic changes of tumor molecular profiling over time.

Consistent with published studies^33,37^, the high recapitulation of the genomic landscape was shown in our study when NSCLC PDX was compared with lung primary tumors. At present, the morphological and genomic fidelities were repeatedly confirmed in a series of PDX models; however, little attention has been paid to the molecular heterogeneity between and within tumors^37^. We found that more than 35 oncogenes were differentially expressed among 3 patients and preserved in the corresponding PDXs. Except for genes that had been shown to be mis-regulated in many cancer types, such as* CDKN2A*, *CCND1*, and *IL6*, we also detected a cohort of genes that had been newly or rarely reported in LC. For example, *CD274* (*PD-L1*) gained wide attention in the last 2 years, and was used in clinical NSCLC immunotherapy^38^, *LCN2* was reported to associate with the epithelial to mesenchymal transition^39^ and radioresistance in lung cancer^40^. Gene co-expression network analysis (http://www.coexpedia.org, http://genemania.org) among these genes indicated a valuable resource for molecular mechanism research of intertumoral heterogeneity. Therefore, clarifying the function of these differentially expressed genes may provide new evidence for personalized treatments.

According to the latest NCCN Guidelines for NSCLC (https://www.nccn.org), ROS1 rearrangement and BRAF V600E mutation were newly incorporated into initial gene testing, and the recommendation for PD-L1 detection was strengthened from 2A to 1. In addition, the treatment of NSCLC patients was also divided into 3 categories based on the gene test results as follows: 1, targeted therapy for patients with gene mutation; 2, immunotherapy for patients with PDL1 ≥ 50% (positive); and 3, patients who did not benefit from targeted and immunotherapy and were returned to traditional radiotherapy and chemotherapy. This indicated that the diagnosis and treatment of NSCLC is moving towards a more individualized and precise direction, which poses an unprecedented challenge for designing an ideal preclinical biological model system to tailor approaches in innovative drug discovery, diagnostic techniques, and pathogenesis research. In our present study, the histological, molecular, and pharmacodynamic analyses of NSCLC PDX models proved that PDX was able to faithfully mirror the phenotypic and genomic features, heterogeneity, and drug response of corresponding patient tumors, which may provide the most clinically relevant tumor models for testing personalized approaches in preclinical settings.

## Conclusions

We have developed a large collection of serially transplantable PDX models for NSCLC. These PDX models authentically recapitulated the features of the primary tumors, including histopathology, gene expression, mutations, DNA copy number alterations, drug sensitivities, and intra- and intertumor heterogeneities. Overall, these patient-derived NSCLC xenograft models provide an excellent platform for preclinical screening of novel cancer therapeutics, discovery of biomarkers, and guidance of individualized cancer therapies.
