# Supplementary material for: Patient-derived non-small cell lung cancer xenograft mirrors complex tumor heterogeneity
Source: Cancer Biol Med. 2021 Feb 15;18(1):184–98. doi: 10.20892/j.issn.2095-3941.2020.0012 (PMC7877179; doi:10.20892/j.issn.2095-3941.2020.0012)
Supplement: Supplementary file 1 [file cbm-18-184-s001.pdf]

Supplementary materials

Table S1 Clinical and pathological characteristics of 75 lung cancer patients and their tumors

Patient ID	Age (years)/ gender	Smoking history (years)	Differentiation	TNM stage	Site	Histological cell type	Tumor size (cm^3)	Metastasis
LC-00158	44/F	N	Moderately	IA	LUL	ADC	1.86	N
LC-00368	70/M	40	Poorly	IIB	RLL	SCC	18.85	N
LC-00536	60/F	N	Poorly	IIIA	RML	ADC	1.00	Y
LC-00666	71/F	N	Moderately	IB	LLL	SCC	31.41	N
LC-00592	62/M	40	Poorly	IIIB	RLL	ADC	126	Y
LC-00234	72/M	50	Poorly	IIB	RLL	SCC	81.73	N
LC-00576	66/M	45	Poorly	IA	RLL	ADC+SCC	2.25	N
LC-00088	52/M	20	Poorly	IB	RUL	SCC	25.13	Y
LC-00453	61/F	N	Poorly	IIIA	RLL	ADC	13.50	Y
LC-00374	76/M	30	Moderately	IB	RLL	SCC	14.14	N
LC-00178	73/M	30	Moderately	IB	RML	ADC	13.50	N
LC-00144	63/M	7	Moderately	IB	RLL	ADC	16.49	N
LC-00507	73/M	50	Poorly	IB	RUL	SCC		N
LC-00083	62/M	N	Poorly	IIIB	RML	SCC	108.00	Y
LC-00022	60/F	N	Moderately	IB	LUL	ADC	5.24	N
LC-00001	59/M	45	Moderately	IIA	LUL	SCC	108.00	N
LC-00827	70/M	50	Poorly	IIA	RLL	SCC	40.00	Y
LC-00877	58/M	35	Poorly	IB	LUL	SCC	32.00	N
LC-00781	61/M	40	Poorly	IIA	RLL	SCC	12.76	Y
LC-00343	59/M	40	Poorly	IIIA	LUL	SCC	12.76	Y
LC-00095	74/M	50	Poorly	IB	RLL	SCC	0.60	N
LC-00304	55/M	30	Poorly	IIIA	RLL	SCC	144	Y
LC-00702	62/M	45		IB	LLL	SCC	6.00	N
LC-00894	67/F	N		IA	RLL	ADC	4.19	N
LC-00010	55/F	N		IB	RUL	ADC	18.00	N
LC-00829	53/F	N		IA	LUL	ADC	4.19	N
LC-00813	66/M	45	Poorly	IIIA	RUL	ADC	65.45	Y
LC-00444	43/F	N		IB	LLL	SCC	13.74	Y
LC-00053	61/F	N			LUL	ADC	7.85	
LC-00700	62/M	30			RLL	SCC		
LC-00831	59/F	N	Moderately	IB	RLL	ADC	7.85	N
LC-00195	51/M	30	Poorly	IIIA	LLL	ADC	144.00	Y

Table S1 Continued

Patient ID	Age (years)/ gender	Smoking history (years)	Differentiation	TNM stage	Site	Histological cell type	Tumor size (cm^3)	Metastasis
LC-00167	52/M	30	Poorly	IIB	RUL	ADC	16.89	Y
LC-00576	62/F	N	Poorly	IA	LUL	ADC	15.08	N
LC-00615	55/F	N		IA	LUL	ADC	0.13	N
LC-00200	52/M	20	Well	IA	LUL	ADC	0.52	N
LC-00193	71/F	0	Poorly	IB	RUL	ADC	5.78	N
LC-00886	74/F	N	Poorly	IIA	RLL	ADC	25.13	Y
LC-00526	62/F	N	Poorly	IV	LLL	ADC	18.85	Y
LC-00080	51/F	N			RUL	ADC		
LC-00349	42/M	30	Well	IIIA	RML	ADC	196.00	N
LC-00100	67/M	30		IA	LLL	SCC	6.78	N
LC-00338	64/M	45	Poorly	IA	RUL	ADC	1.00	N
LC-00520	37/M	N	Poorly	IIIA	LUL	ADC	3.14	Y
LC-00065	52/M	30	Poorly	IB	LUL	SCC	21.99	N
LC-00516	70/F	N	Poorly	IIA	LUL	ADC	1.05	Y
LC-00157	51/F	N	Moderately	IB	RLL	ADC	13.04	N
LC-00327	56/M	40	Poorly	IIA	RLL	ADC		Y
LC-00111	88/M	N	Moderately	IB	RUL	SCC	6.00	N
LC-00798	60/F	N	Moderately	IB	RML	ADC	18.85	N
LC-00441	46/F	N	Moderately	IA	RLL	ADC	2.60	N
LC-00442	44/F	N	Poorly	IA	RUL	ADC	7.81	N
LC-00921	79/F	N	Poorly	IB	LUL	SCC	18.00	N
LC-00428	52/M	N	Moderately	IB	RUL	ADC	6.05	N
LC-00339	70/M	40	Moderately	IIB	RLL	ADC	9.42	Y
LC-00112	59/M	30	Poorly	IB	RML	ADC	3.61	N
LC-00597	71/M	50	Poorly	IB	RLL	SCC	9.42	N
LC-00335	75/M	30	Poorly	IIB	LLL	ADC	31.41	Y
LC-00185	66/F	N	Moderately	IIA	RUL	ADC	1.05	Y
LC-00333	71/F	N	Moderately	IA	LUL	ADC	1.69	N
LC-00198	56/M	40		IA	RUL	ADC	0.29	N
LC-00223	59/M	2	Moderately	IA	RLL	ADC	4.54	N
LC-00844	47/M	18	Moderately	IA	LUL	ADC	0.1	N
LC-00910	46/M	10		IA	LUL	LCNEC	0.03	N
LC-00213	44/F	N	Poorly	IA	RLL	ADC	5.6	N
LC-00095	52/M	N	Moderately	IA	RUL	ADC	0.75	N

Table S1 Continued

Patient ID	Age (years)/ gender	Smoking history (years)	Differentiation	TNM stage	Site	Histological cell type	Tumor size (cm^3)	Metastasis
LC-00192	55/F	N	Poorly	IA	LUL	ADC	0.31	N
LC-00214	70/M	50	Poorly	IA	LLL	ADC	0.47	N
LC-00125	53/F	N	Moderately	IA	RML	ADC	2.25	N
LC-00649	76/F	N	Moderately	IA	LUL	ADC	1.14	N
LC-00294	52/M	12		IA	RUL	ADC	0.7	N
LC-00820	39/M	15		IA	RUL	ADC	0.03	N
LC-00879	59/M	10		IA	RUL	ADC	1.57	N
LC-00078	70/F	N	Moderately	IA	RUL	ADC	1.44	N
LC-00621	60/F	N	Moderately	IA	LLL	ADC	2.82	N

TNM, tumor node metastasis; ADC, adenocarcinoma; SCC, squamous cell carcinoma, LCNEC, large-cell neuroendocrine carcinoma; LUL, left upper lobe; RLL, right lower lobe; RML, right middle lobe; LLL, left lower lobe; RUL, right upper lobe. The blank space indicates no information.

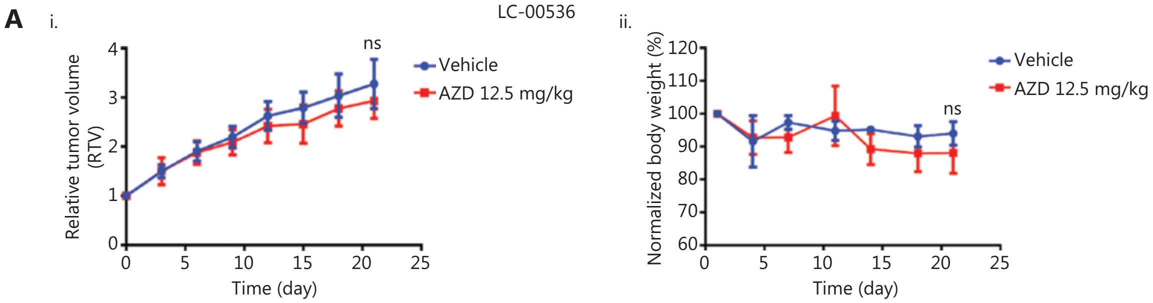

Figure S1 Efficacy validation of an investigational new drug. Changes in Ai, relative tumor volume and Aii, body weight after patient-derived xenograph mice of LC-00536 were treated with AZD4547 for 21 days.

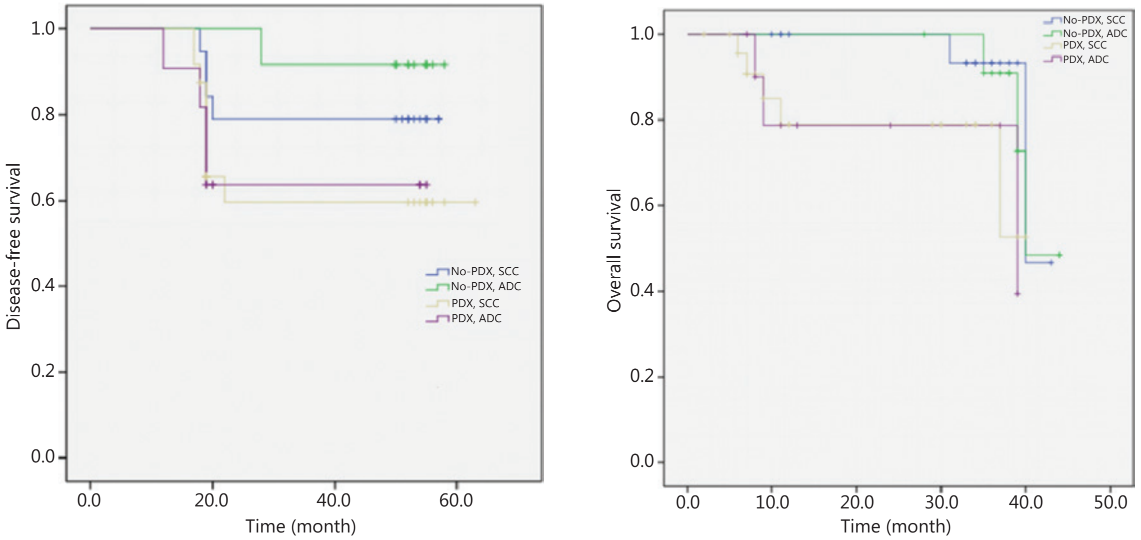

Figure S2 Disease-free survival and overall survival of non-small cell lung cancer patients according to the engraftment status and histological cell type of their patient-derived tumor xenografts.
